# Recent advances in understanding oogenesis: interactions with the cytoskeleton, microtubule organization, and meiotic spindle assembly in oocytes

**DOI:** 10.12688/f1000research.13837.1

**Published:** 2018-04-17

**Authors:** Florence L. Marlow

**Affiliations:** 1 Department of Cell Developmental and Regenerative Biology, Icahn School of Medicine at Mount Sinai, New York, New York, USA

**Keywords:** meiosis, microtubules, actin, oocyte, oogenesis

## Abstract

Maternal control of development begins with production of the oocyte during oogenesis. All of the factors necessary to complete oocyte maturation, meiosis, fertilization, and early development are produced in the transcriptionally active early oocyte. Active transcription of the maternal genome is a mechanism to ensure that the oocyte and development of the early embryo begin with all of the factors needed for successful embryonic development. To achieve the maximum maternal store, only one functional cell is produced from the meiotic divisions that produce the oocyte. The oocyte receives the bulk of the maternal cytoplasm and thus is significantly larger than its sister cells, the tiny polar bodies, which receive a copy of the maternal genome but essentially none of the maternal cytoplasm. This asymmetric division is accomplished by an enormous cell that is depleted of centrosomes in early oogenesis; thus, meiotic divisions in oocytes are distinct from those of mitotic cells. Therefore, these cells must partition the chromosomes faithfully to ensure euploidy by using mechanisms that do not rely on a conventional centrosome-based mitotic spindle. Several mechanisms that contribute to assembly and maintenance of the meiotic spindle in oocytes have been identified; however, none is fully understood. In recent years, there have been many exciting and significant advances in oogenesis, contributed by studies using a myriad of systems. Regrettably, I cannot adequately cover all of the important advances here and so I apologize to those whose beautiful work has not been included. This review focuses on a few of the most recent studies, conducted by several groups, using invertebrate and vertebrate systems, that have provided mechanistic insight into how microtubule assembly and meiotic spindle morphogenesis are controlled in the absence of centrosomes.

## Introduction

During meiosis, the genome is duplicated without immediate cytokinesis, resulting in a cell with twice the number of chromosomes normally found in somatic cells, a 4N cell. The meiotic divisions will eliminate these excess chromosomes to produce the haploid gametes, with half the number of chromosomes normally found in somatic cells that are required for sexual reproduction. In males, the meiotic divisions yield four “equivalent cells”, sperm, with single copies of each chromosome; however, in females, through unequal cytoplasmic divisions, a single large oocyte and tiny non-functional polar bodies are produced. Prior to equal chromosome segregation into the asymmetric daughter cells, the nucleus, which often occupies a central position in early oocytes, is moved toward the cortex just before GVBD or NEBD. In general, the region occupied by the oocyte nucleus prior to GVBD (NEBD) is defined as the animal pole; this is the side of the cell where the small polar bodies will be eliminated. Defects in meiosis lead to aneuploid gametes, sperm, or oocytes with the wrong number of chromosomes, which can lead to miscarriage, birth defects, and infertility. Given that meiotic division in females generates only one viable cell that when fertilized will produce an embryo, it may seem somewhat surprising that there appears to be no mechanism to selectively retain specific chromosomes in the oocyte (for example, those free of mutations). Instead, with the exception of the few reported selfish genes, the process seems to be stochastic, a trade-off that possibly facilitates evolution as the selection of specific chromosomes might hinder the acquisition of advantageous mutations in response to selective pressures. Early in oogenesis, the centrosomes are lost, leaving these enormous cells with unique challenges during karyokinesis, namely to navigate chromosome separation and elimination to achieve the haploid state without the aid of a centrosome-based apparatus. Three pathways have been found to operate in cells without centrosomes: the Ran/Importin pathway, the chromosome passenger complex (CPC) pathway, and the Augmin pathway; however, these pathways are not fully understood, and the extent to which each of these pathways contributes to spindle assembly and meiotic division within and across species is not known. This review will highlight recent advances toward understanding the cellular and molecular mechanisms that contribute to regulating the position of the nucleus, acentrosomal microtubule assembly, and morphogenesis of the meiotic spindle in oocytes. A unifying theme emerging from studies of the oocytes of model organisms is that these processes rely on mechanisms that involve facilitating or limiting interactions with the actin and microtubule cytoskeletons and associated proteins to generate spatially restricted activities within these large meiotic cells.

## Roles for actin in positioning the nucleus, the spindle, and gathering chromosomes

In the oocytes of many organisms, the nucleus moves from a central to a cortical position prior to or around the time of the meiotic divisions. In *Caenorhabditis elegans* and some mammalian oocytes, such as the blue fox, oocyte nucleus translocation occurs in response to maturation hormone ^1– 3^. However, such translocation has not been observed in mouse oocytes matured *in vitro*, where the nuclear envelop has been observed to break down in a central position upon stimulation with maturation factors ^4^. In this context, later spindle position is not disrupted, indicating that if redistribution of the nucleus contributes to spindle position, it is not an essential prerequisite. It is unclear whether this difference in nuclear relocation between the oocytes of mice and foxes reflects the possibility that the mouse oocytes were cultured and the fox oocytes were observed *in situ*. Evidence that the somatic follicle may influence nucleus position and later spindle assembly in mouse oocytes was provided by work from Barret and Albertini in which eccentric nuclei were observed in matured cumulus cell-enclosed oocytes ^5^. In that context, cortical positioning of the nucleus and spindle was observed to be both contact- and actin-dependent, as latrunculin A treatment resulted in centrally positioned nuclei and spindles ^5^. Consistent with a role for actin in regulating cortical spindle position, mutations disrupting the actin-binding protein, Formin 2 ^6, 7^, Cdc42 ^8^, or Myosin II ^9^ cause central spindle and aneuploidy in mouse oocytes. Moreover, earlier work showed that meiosis I chromosomes move to the cortex in an actin-dependent manner ^7, 10, 11^, and in mouse and *Xenopus* oocytes, cytochalasin B treatment causes spindle defects ^12– 14^. Other studies of mouse oocytes implicated an actin meshwork in spindle migration based on the observation of actin meshworks using Phalloidin and green fluorescent protein (GFP) reporters of actin-binding proteins ^9, 15, 16^. However, how the meshwork would contribute to spindle migration was unclear because different manifestations of the meshwork were observed, possibly because of the different reporters used between studies. Thus, there are varied models for how the actin meshwork might facilitate spindle transport; these models included transport via MyoII, the major force generator for contractile microfilaments ^9^, MyoII-mediated force generation, and pushing via actin polymerization ^15^. The absence of a clear picture of filament organization with respect to cytoskeletal polarity and the lack of evidence supporting the association of specific myosin motors with the spindle left the meshwork-mediated mechanism of spindle migration unresolved.

Although microtubules are the cytoskeletal system that comes to mind when considering spindle and chromosome segregation, actin filaments have recently been found to be a conserved component of spindles ^17^, including in human oocytes. Studies in mice aimed toward uncovering the function of this spindle-associated actin utilized actin poisons and stabilizing drugs as well as analysis of *formin2* mutants revealed a role for actin, more specifically for actin dynamics, in promoting chromosome separation on both meiosis I and meiosis II spindles ^17^. Interestingly, this activity was important for the assembly of Kinetochore-fibers (K-fibers), microtubule bundles that together with microtubule-associated proteins generate forces underlying chromosome separation ^17^. Using inhibitors and overexpression strategies, the authors showed that actin density and K-fiber density were similarly changed in response to conditions that increase or decrease actin. Although it is clear that actin association with the spindle is conserved and is required for accurate chromosome segregation in the mouse and other species, how actin dynamics regulate K-fiber density is not fully understood.

Notably, in starfish oocytes, microtubules are not adequate to recruit chromosomes to the spindle ^18^. In these oocytes, actin polymerization is required to collect the chromosomes. Upon nuclear envelop breakdown (NEBD), an actin network assembles in the vicinity of the nucleus and is required for effective chromosome capture, as actin-depolymerizing poisons lead to aneuploidy ^18, 19^. Interestingly, assembly of this network is triggered by NEBD, and time-lapse analyses indicate that actin nucleation is a chromatin-mediated process, as beads with DNA but not beads alone could induce actin patch formation ^18^. Isotropic and uniform contraction of the network was proposed to attain directionality via tethering to the cortex, which would result in cortical transport of chromosomes entrapped within the meshwork ^19^. Although the actin meshwork had been described as contractile, it was not clear whether the process was myosin-mediated or instead relied on actin depolymerization as has been observed during closure of the cytokinetic ring ^20– 22^. In very recent follow-up work, using the chromosomes as endogenous probes to assess the contractile behavior of the network, Bun and colleagues confirmed that the network contracted in a uniform manner ^23^. To investigate actin dynamics associated with contraction, the authors monitored fluorescent actin reporters and, in pulse chase-like experiments, observed actin polymerization initiating from the remnants of the nuclear envelope at the periphery such that the void associated with contraction is replenished by new filaments ^23^. This observation raised the possibility that assembly of the new network provides force to push the entrapped chromosomes to the cortex. However, ablation and stabilization studies indicated that the forces are throughout the network rather than driven by actin assembly at the periphery ^23^. Surprisingly, overexpression and inhibitor experiments to activate or block MyoII did not disrupt chromosome movement or contraction rate and thus excluded a MyoII-dependent process ^23^. Moreover, the absence of detectable vesicles in the vicinity of the nucleus and inhibition of MyoVb indicated that directed transport was unlikely to contribute ^23^. In contrast, stabilizing actin filaments provided evidence that disassembly drives contraction rates. Accordingly, pan inhibition of formins using a small-molecule inhibitor of the FH2 domain essential for formin self-interaction and actin nucleation similarly impaired network contractility ^23^, implicating a formin in regulating actin dynamics that provides forces to promote chromosome gathering during meiosis. Key questions that remain to be resolved include identifying the relevant formin and its regulators and determining whether and to what extent actin disassembly-based mechanisms are used to move and deliver cargo to the cortex in oocytes.

In 2013, a novel meiotic cell-specific nuclear actin-bundling Kinesin 3, Nabkin, was identified among known actin-binding proteins and regulators in actin complexes purified by exposing isolated *Xenopus* nuclei to a phalloidin matrix ^24^. In early oocytes, prior to germinal vesicle breakdown (GVBD) or NEBD, Nabkin resides in the nucleus of *Xenopus* oocytes ^24^, which is devoid of microtubules ^25^; thus, Nabkin is unlikely to interact with microtubules at this stage. After nuclear translocation to the animal pole and GVBD, Nabkin is exposed to microtubules and localizes to the transient microtubule array, a network that gathers the chromosomes and the meiotic spindle ^24^. In addition to *de novo* interactions with microtubule structures, Nabkin was observed to maintain its associations with actin structures at the animal pole. These actin structures are involved in the asymmetric divisions that produce the polar bodies, and, based on antibody interference studies, this process requires Nabkin interaction with actin ^24^. How Nabkin selectively associates with meiotic spindle microtubules after NEBD and its functions there remain unclear. The recent findings in the mouse of the connection between spindle-associated actin and K-fibers ^17^ raise the interesting possibility that Nabkin is the missing link between actin and K-fibers. Moreover, like Nabkin, the related Kinesin 14 associates with the mitotic spindle and contractile F-Actin apparatus in human somatic cells ^24^. Thus, it is possible that Nabkin and Kinesin 14 fulfil similar roles in these distinct cell types, but this remains to be determined.

## Spectraplakin functions in nuclear position, actin tethering, and microtubule organization

**Figure 1.**
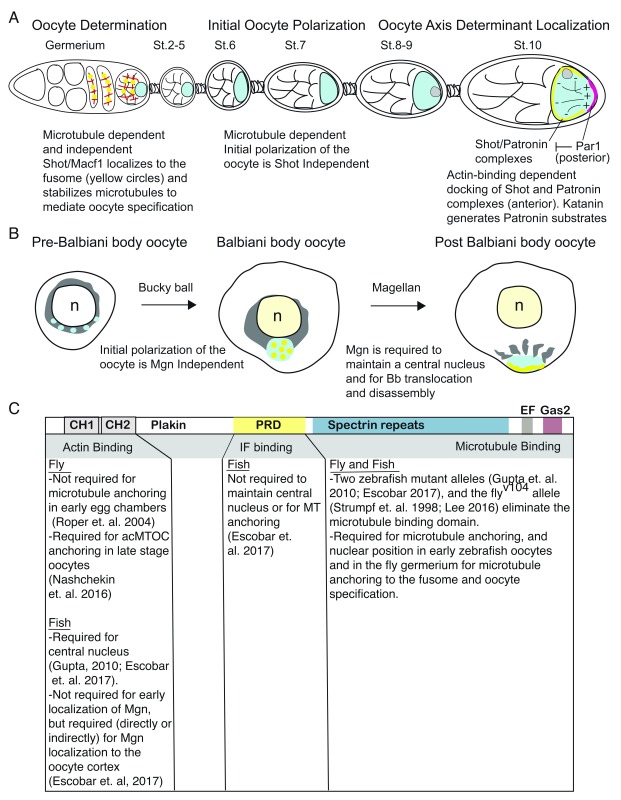
Schematic depicting functions of the Spektaplakin Macf1 to oogenesis in *Drosophila* and zebrafish. ( **A**) Drawing depicting *Drosophila* oogenesis and the stages and processes mediated by Shot/Macf1. ( **B**) Drawing depicting selected stages of zebrafish oogenesis to highlight Mgn/Macf1-dependent processes. ( **C**) Schematic depicting Macf1 functional domains and the contribution of each functional domain to Macf1-mediated processes in *Drosophila* and zebrafish. acMTOC, acentrosomal microtubule-organizing center; IF, intermediate filament; Mgn, Magellan; MT, microtubule; PRD, proline-rich domain; St., stage.

Shot, also known as Kakapo, Macf1, and Magellan, is a Spectraplakin with microtubule and actin interaction domains that has cross-linking activity and is essential for normal oogenesis ( Figure 1). Analyses of mutants and germ-line clones disrupting *Drosophila shot/macf1* revealed its crucial role in oocyte specification ( Figure 1) ^26, 27^. In the *Drosophila* ovary, a single oocyte is specified from 16 interconnected cells that are called cystocytes. This process involves the transfer of essential contents, including cellular organelles, such as mitochondria and centrosomes, along with proteins and RNAs through a process that, as studies of mutants and ovaries treated with cytoskeleton poisons revealed, involves molecular motors and microtubule-dependent as well as microtubule-independent mechanisms ^28– 33^ and the fusome localized protein, Shot/Macf1 ^26^ ( Figure 1A). In *shot* mutant clones, deficits in microtubule anchoring to the fusome and organization reduced centrosome numbers, and oocyte specification deficits revealed Shot’s essential contribution to early oogenesis ^26^. The observation that Shot co-localized with acetylated microtubules on the fusome of meiotic cysts, even after treatment with microtubule depolymerizing agents, but not with actin in the ring canals, and that acetylated microtubules were reduced in *shot/macf1* mutant clones hinted that microtubules, specifically microtubule stability, requires Shot/Macf1 ^26^. This notion was further supported by the observation that mutant alleles lacking the actin-binding domain could support oocyte specification and fertility; thus, Shot/Macf1 was proposed to mediate centrosome migration and oocyte specification by a mechanism that involves protection of a subset of microtubule poison-resistant microtubules, specifically the acetylated microtubules, associated with the fusome ^26^. Later, zebrafish *magellan/macf1*, discussed below, was similarly shown to be essential for microtubule anchoring in early oocytes and fertility ^27^ ( Figure 1B).

Recently, new alleles disrupting *Drosophila shot* were discovered that could support *shot* functions in oocyte specification and thus allowed *shot* functions in stages after oocyte specification to be examined ^34, 35^ ( Figure 1A, C). When fluorescent reporters were used to examine Shot protein localization in *Drosophila* oocytes, Shot protein was shown to accumulate in anterior lateral regions but not the posterior pole ^35^. Because *shot* RNA is more broadly expressed than the protein, this spatially restricted pattern seems to be achieved by restricting where *shot* RNA is translated and by limiting the diffusion of Shot protein through its interactions with the actin cytoskeleton in anterior regions ^35^. Specifically, an actin-binding and Par1-dependent mechanism is likely, as residues within Shot’s actin-binding domain are required to localize Shot, and the protein ectopically accumulates in posterior regions of *par1* mutant oocytes ^34, 35^. Because no evidence for Shot/Macf1 phosphorylation was detected, Par1 likely modifies the structure of the posterior cortex or cortical proteins that localize there to prevent Shot/Macf1 accumulation in the posterior region of *Drosophila* oocytes ^34^ ( Figure 1A). Although no deficits in the actin cytoskeleton were detected in *shot* mutant oocytes, fewer microtubules occupied anterior regions of later-stage oocytes, indicating that Shot promotes regional microtubule assembly or locally stabilizes existing microtubules ^34, 35^. The co-localization observed between Shot and Patronin and deficits in microtubules and Patronin protein in anterior regions of *shot/macf1* mutant oocytes led to a model whereby Shot binds to and recruits the microtubule minus end binding protein Patronin to the anterior pole to establish a platform for localized microtubule assembly ^34^ ( Figure 1A). Consistent with this model, the actin-binding domain of Shot is required for anchoring of non-centrosomal microtubule-organizing centers (MTOCs), and defective anchoring of acentrosomal MTOCs, as occurs in *shot* mutants, leads to microtubule disorganization ^34, 35^. Like Patronin, Katanin, a microtubule-severing protein, localizes to the anterior cortex ^34^ ( Figure 1A). The restricted localization and proximity of these factors in *Drosophila* oocytes provide a means to spatially control microtubule assembly with substrates for Patronin stabilization and assembly generated by Katanin in a manner that is independent of γ-Trc nucleation and centrioles. Additional evidence for a mechanism whereby Shot/Macf1 functions at MTOCs includes the observation that Shot/Macf1 localizes to the ectopic MTOCs that form in mutants with hypermorphic alleles of the actin nucleator *cappuccino* ^35^. Taken together, these recent studies provide evidence for a spatially restricted, actin-tethered system to generate MTOCs independent of centrosomes. It will be interesting to determine whether Spektraplakins contribute to meiotic spindle organization or position during meiotic divisions as a component of, or in a manner analogous to, the actin meshwork of mouse and starfish oocytes discussed above. Determining the identity and nature of the (likely cortical) targets of Par1 modification that render the posterior cortex restrictive to Shot/Macf1 or, alternatively, factors that render the anterior region permissive for Shot/Macf1 association will provide insight into how the oocyte maintains regional identity.

Like *Drosophila* Shot/Macf1, the corresponding zebrafish protein, called Magellan, localizes to the oocyte cortex ^36^, and is required to anchor microtubules to the cortex, to maintain a central nucleus and to facilitate translocation of the Balbiani body from a perinuclear position to the vegetal cortex, where it is disassembled ^27^ ( Figure 1B). Actin binding does not appear to be required for perinuclear localization of Magellan/Macf1 to the Balbiani body of early oocytes; however, an intact actin-binding domain is required for translocation of the Balbiani body toward the cortex ^36^. Therefore, actin binding may be required indirectly or directly for Mgn association with the vegetal cortex, since Balbiani body cargo are not delivered in *mgn* mutants predicted to encode for proteins that partially disrupt the actin-binding domain ^36^ ( Figure 1B, C). Likewise, the intermediate filament association domain is dispensable for Mgn/Macf1 localization to the Balbiani body and for Mgn/Macf1 functions in nuclear position, Balbiani body translocation, and disassembly ^36^. Interestingly, the lack of detectable Mgn/Macf1 protein in early oocytes of at least one mutant allele predicted to encode a truncated protein without the microtubule-binding domain indicates that the mutant proteins may be inherently unstable or that microtubule binding is important for localization to the Balbiani body, which in turn may affect the stability of these truncated Mgn/Macf1 proteins ^27, 36^. Whether eventual localization of Magellan/Macf1 protein to the oocyte cortex first requires its localization to the Balbiani body is not known because Magellan protein localization in Bucky ball ( *buc*) mutants, which lack Balbiani bodies, has not been reported. However, based on epistasis analysis, it appears that concurrent loss of *buc* and *magellan* produces additive phenotypes, oocytes with acentric nuclei and lacking a Balbiani body core marked by Buc protein and densely populated mitochondria ^36^. Therefore, Macf1 regulates nuclear position by a mechanism that is independent of impaired Balbiani body dynamics. Whether detachment of microtubules from the cortex, as occurs in *magellan* mutants lacking the microtubule-binding domain, still occurs in the actin-binding deletion alleles or in the absence of Buc and the Balbiani body has yet to be reported. The mechanism by which Magellan/Macf1 maintains a central nucleus is unclear; however, since mutant alleles that disrupt the microtubule-binding domain and alleles that disrupt the actin-binding domain both result in nuclear displacement, it seems likely that this phenotype involves bridging the cellular space between the nuclear envelope and the cell cortex through interactions with both the actin and the microtubule cytoskeleton or associated factors that may provide balancing forces to stabilize the central position of the nucleus. The molecular factors and relevant interactions remain to be determined.

## Coordinating spatial cues to organize the meiotic spindle without centrosomes

The importance of chromosomal cues to the organization of the meiotic spindle in oocytes, which, unlike mitotic cells, lack centrosomes and tend to be large cells, has been appreciated for more than a decade. In the absence of centrosomes, the chromosomes emerged as a potential catalyst of spindle assembly by a mechanism thought to involve microtubule nucleation or capture. Three pathways to organize the spindle without centrosomes have been defined: two involving chromatin capture and a third that is thought to bias elongation toward the poles. In the Ran-Importin pathway, Ran-GTP liberates Importinα/β-bound spindle assembly factors, including nucleating factors and XCTK2/Kinesin 14. In this pathway, XCTK2/Kinesin 14 spindle interaction and microtubule anchoring occur near the chromosomes where Ran-GTP concentration is high and are prevented by interaction with importinα/β near the spindle poles where Ran-GTP concentration is low ^37– 40^. The Augmin pathway is thought to stimulate spindle morphogenesis by recruiting γ-tubulin to existing microtubules and promoting their elongation ^41– 43^. Consistent with this activity, interference with Augmin in *Xenopus* and *Drosophila* diminishes nucleation of microtubules associated with the spindle ^44, 45^. The observation that Augmin complex components are enriched at the poles in *Drosophila* oocytes, but not S2 (cultured *Drosophila* somatic cells), and that Augmin components turn over more slowly based on fluorescence recovery after photobleaching (FRAP) experiments has led to a model whereby in oocytes Augmin activity is biased toward the poles ^45^. The third pathway, the CPC pathway, stabilizes microtubules and promotes spindle assembly in *Xenopus* egg extracts and in zebrafish and *Drosophila* oocytes and early embryos ^46– 52^. Although the CPC, a key complex in spindle morphogenesis, and in particular the kinase subunit Aurora A, which associates with chromatin and is essential for spindle I assembly, was implicated as a component of a chromosome-based cue system ^48, 51, 53, 54^, it was not clear how such spatial information would be transmitted from the chromosomes to promote spindle organization or dynamics. In addition, it was not clear how independently each of these organizing systems operates within cells or species. Indeed, interference with the Ran pathway in *Drosophila* and mouse oocytes did not abolish assembly of the meiosis I spindle, and although complete loss of CPC function blocks spindle microtubules altogether, partial loss of function significantly delays assembly ^48, 51, 55, 56^, indicating that some of these mechanisms may be redundant or that additional unknown mechanisms are deployed in cells lacking centrosomes.

## Bundling motors and meiotic spindle morphogenesis

The microtubule regulator Mini spindles (Msps) (also known as XMAP215/TOG) localizes to the poles of the meiotic spindle, and mutations disrupting *msps* cause tripolar spindles to form in metaphase I oocytes of *Drosophila* ^57^. Similarly, depletion of two plus end-directed microtubule-associated kinesin motors, *subito* ( *sub*) or *kinesin 6*, and *non-claret disjunctional* ( *ncd*), first observed in 1929 and later shown to be a Kinesin 14, disrupts spindle morphogenesis ^58– 63^. In the case of *ncd* mutants, spindle polarity defects were associated with failure to cluster microtubule minus ends and failure to localize Msps to the spindle poles ^57, 61, 64^. Based on the observation that the localization of Msps, but not D-TACC ( *drosophila* transforming, acidic, coiled-coil containing), another spindle pole and centrosome-localized protein, was disrupted in *ncd* mutants, a model was put forth wherein the minus end-directed motor Ncd transports Msps to the spindle pole where it interacts with and is anchored by its binding partner, D-TACC, which localizes earlier by a mechanism that does not require Ncd ^57^. Moreover, based on the observations that *d-tacc* mutant oocytes have similar spindle defects and that Msps localization requires D-TACC, interaction between these two proteins specifically at the spindle pole could then stabilize the bipolar meiotic spindle ^57^. The essential role of Sub/Kinesin 6 for bundling of interpolar microtubules was demonstrated through mutant and structure function rescue assays ^63^. Studies of truncated versions of Sub/Kinesin 6 revealed unique activities of the N-terminus in microtubule bundling near the chromosomes, of the motor domain in central spindle organization, and of the C-terminal domain in binding to the plus ends of the central spindle ^63^. In that work, Sub/Kinesin 6 was shown to bundle anti-parallel microtubules by a mechanism thought to be triggered by a diffusible factor released or activated upon NEBD rather than by activation or capture of microtubules through contact with the chromosomes, a mechanism indicated by earlier studies, because Sub interacts with microtubules only after NEBD ^61, 63, 65, 66^. Based on its localization and activity in microtubule assembly assays in other organisms, Ran was postulated to be a candidate factor emanating from the chromosomes to set up a gradient capable of triggering spindle assembly ^67, 68^; however, among the components examined, only CPC was shown to be required for meiosis I spindle formation in *Drosophila* ^48, 51, 55, 56^. Therefore, at least two key questions remained to be addressed: what is the identity of the putative factor that establishes the spindle-organizing region and promotes chromatin-mediated spindle assembly, and what is responsible for organizing the bipolar spindle in the absence of centrosomes?

**Figure 2.**
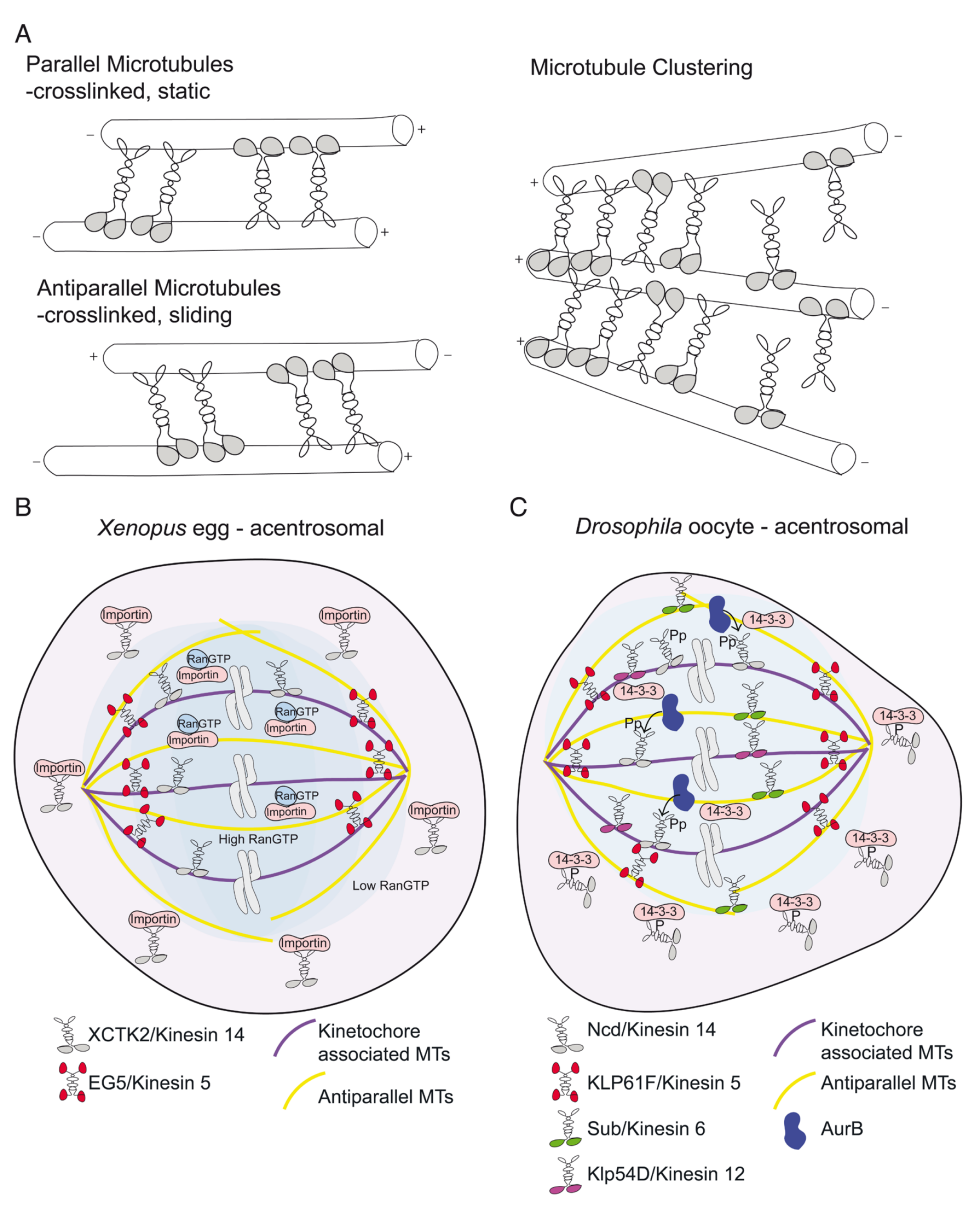
Activities of microtubule-organizing kinesins and comparison of two pathways mediating assembly of acentrosomal spindles. ( **A**) Illustrations depicting how Kinesins can crosslink microtubules to promote the sliding and clustering necessary for sorting of anti-parallel microtubules and spindle assembly and stability. ( **B**) Drawing depicts the Ran-Importin pathway described for *Xenopus* egg extracts. Kinesin 14 interacts with Importin and is inhibited from interacting with microtubules. Importin inhibition is alleviated near the chromosomes, where Ran-GTP concentration is high. Ran-GTP association with Importin allows Kinesin 14 to associate with kinetochore-bound microtubules. Balancing forces from Kinesin 5 stabilize the bipolar spindle. ( **C**) Illustration of a pathway for meiotic spindle assembly in *Drosophila* oocytes. Kinesin 14 interacts with 14-3-3 and is inhibited from interacting with non-spindle microtubules. Near the chromosomes, Aurora B phosphorylation dissociates 14-3-3 and unmasks the repressed microtubule-organizing activity of Kinesin 14. As in *Xenopus* extracts, balancing forces from Kinesin 5 and two additional Kinesins promote assembly and stabilization of the bipolar spindle. AurB, Aurora B; Klp61F, kinesin-like protein 61F (Kinesin 5); MT, microtubule; Ncd, non-claret disjunctional; Sub, subito; XCTK2, *Xenopus* COOH-terminal kinesin 2 (Kinesin 14).

Unique structural features and activities of individual Kinesin classes and studies of mitotic cells and *in vitro* assays indicated that the problem of spindle organization was more interesting and complicated than simple motor-mediated transport along microtubules ^69– 73^. Antagonistic actions of Kinesins had been observed in *Drosophila*, mammalian, and yeast cells ^74– 76^. A few years later, *in vitro* assays using fluorescently labeled microtubules and tagged Kinesin 5 (Eg 5), long suspected of being involved in microtubule manipulation because of its unique bipolar structure with motors at both ends of the stalk, showed that Kinesin 5 could slide microtubules in an orientation-dependent manner ^77^. Similarly, photoactivation of a light-inducible Kinesin5paGFP and photobleaching of labeled microtubules obtained from *Xenopus* egg extracts provided evidence that both Kinesin 5 and microtubules were mobile in the middle of the spindle and that Kinesin 5 movement toward and concentration at the spindle poles was Dynein-dependent, as p150 could block this ^78^. Together, these studies indicated that Kinesin 5 slides parallel microtubules but locks anti-parallel microtubules, providing evidence for a model wherein opposing motors could supply unique activities during spindle morphogenesis. For anti-parallel microtubules, the forces associated with transport cause the microtubules to slide in opposite directions, but when the microtubules are oriented the same way, switching of the motors between microtubules has been proposed to generate opposing forces that effectively lock the microtubules in place ^79^ ( Figure 2A). Cumulatively, these and other studies of Kinesin activities in mitotic cells and *in vitro* systems provided support for models in which selective sorting and entrapment of spindle microtubules could be accomplished by balanced but opposing forces produced by distinct molecular motors. Moreover, it opened up questions about whether localized and selective activity of molecular motors might contribute to meiotic spindle assembly and, if so, how such activities would be regulated, particularly in cells lacking centrosomes, such as oocytes.

The functions of spindle proteins that are required specifically in meiotic cells, either because these genes are expressed only in meiotic cells or because the genes are expressed in and localized to the spindle poles of both mitotic and meiotic cells but act redundantly with other mitotic spindle components, can be studied with traditional mutagenesis approaches as long as they are viable to reproductive stages. In contrast to those proteins, Kinesin 5 (Klp61F in *Drosophila*) localizes to centrosomal and acentrosomal spindle poles in *Drosophila* and mammalian cells ^80– 82^. In mitotic cells, Kinesin 5 prevents the centrosomes from collapsing ^77^, and thus its functions in meiotic spindle morphogenesis could not be determined. To circumvent the mitotic requirement for Klp61F/Kinesin 5, interference strategies were applied in mammalian oocytes, and, more recently, in *Drosophila*, a short hairpin RNA (shRNA) interference strategy was used to target *klp61f* RNA specifically in meiotic cells ^80^. As in mitotic cells, interference with Kinesin 5 using the inhibitor monastral caused a monopolar meiotic spindle phenotype in mammalian oocytes ^81, 82^. However, unlike in mammalian oocytes exposed to monastral, bipolar but asymmetric meiotic spindles were observed in *kinesin 5/klp61f*-depleted *Drosophila* oocytes ^80^. Notably, the more severe phenotypes observed upon simultaneous interference with *kinesin 5/klp61f* and *kinesin 6/sub*, which, like Kinesin 5, bundles anti-parallel microtubules, provided evidence that although individual Kinesins may have similar bundling activities, they can uniquely contribute to meiotic spindle morphogenesis ^80^. Despite the difference in spindle morphology defects of meiotic and mitotic cells depleted of *kinesin 5/klp61f*, both the spindle collapse phenotype observed in mitotic cells and the asymmetric spindle phenotypes of *kinesin 5/klp61f* oocytes appear to be Kinesin 14/Ncd-dependent, as the spindle defects were suppressed by simultaneous depletion of both proteins ^80^. Further highlighting the complexity of meiotic spindle morphogenesis, when similar depletion assays were used, aspects of the meiotic spindle defects were shown to depend on another kinesin, Kinesin 12, and the microcephaly-associated protein, ASP ^80^.

In mitotic cells, ASP localizes to minus ends and to mitotic spindle poles by a mechanism that depends on Kinesin 5/Klp61f ^83^. In that context, cell culture-based activity assays demonstrated that Asp bundles minus ends to other microtubules within the spindle, and at the spindle pole, and is thought to act by an Ncd-independent mechanism based on the intact localization of Asp-GFP to mitotic spindles of Ncd RNA interference (RNAi)-depleted S2 cells and failure of Asp and Ncd to compensate for one another in depletion and rescue studies in cultured *Drosophila* somatic cells ^83^. Kinesin 5/Klp61f counteracts forces generated by Kinesin 14/Ncd, a plus end-bundling protein, on both meiotic and mitotic spindles; however, it counteracts the activity of Asp, a minus end-bundling protein, only on meiotic spindles but not mitotic spindles. This differential effect may be due to technical differences; however, because these distinct functions are observed within the same organism, they cannot be attributed simply to species differences. Instead, it may reflect differences between meiotic and mitotic spindle composition, including (obviously) the absence of centrosomes in oocytes. Centrosomes serve as a source of new microtubules and associate with the mitotic spindle by a mechanism that RNAi and inhibitor studies indicate depends on Asp-mediated crosslinking and Dynein activity but not on Kinesin 14/Ncd ^83– 86^. Taken together, these comparisons suggest that differential use of molecular motors seems to allow the meiotic spindle of oocytes to supply forces or activities that are supplied by the centrosome in mitotic cells. How these activities are spatially and temporally restricted remains an open question.

## Releasing the brakes: liberation from 14-3-3 by the chromosome passenger complex kinase Aurora B

14-3-3 family members are highly conserved proteins that are best characterized for their roles in regulating phosphorylation and mitogen-activated kinase pathways ^87^. Among the molecules that they bind to and regulate are several key signaling proteins, including kinases involved in cell cycle control ^88– 93^. The lack of intrinsic enzymatic activity and identifiable targeting motifs led to models of 14-3-3 action whereby binding interferes with any functional activity proximal to the 14-3-3 interaction domain of the target protein ^87^. Because of their mode of action and roles in signal transduction and cell cycle, 14-3-3 proteins are compelling candidates for involvement in oogenesis. Indeed, 14-3-3 proteins have been shown to play important roles in spindle morphogenesis in mice and *Drosophila* ^94, 95^. In mice, 14-3-3 η localizes to the spindle of metaphase I and II oocytes, and morpholino depletion causes spindle defects by a mechanism that has not been determined ^94^. In *Drosophila*, another 14-3-3 protein promotes MTOC formation in egg chambers and is required for oocyte specification ^95^. Evidence for deficits in MTOC formation include deficits in the localization of mini spindles, a microtubule-associated protein that normally localizes to sites of microtubule nucleation ^95^. Because *14-3-3ε* mutants do not specify oocytes, its potential roles in meiotic spindle morphogenesis were not known.

To circumvent the lack of oocytes in *14-3-3ε* mutants, Beaven and colleagues used an RNAi depletion approach to interfere with 14-3-3ε function in oocytes and found a requirement for 14-3-3ε for bipolar spindle formation based on Tubulin staining and the abnormal localization of the microtubule regulator Msps, also known as XMAP215/Tog ^96^. The similarity between the spindle phenotypes of *14-3-3ε* depleted oocytes and metaphase I oocytes depleted of *msps* provided evidence that the *14-3-3ε* spindle deficits can be explained by defects in Msps regulation ^57, 96^. The deficits in localizing proteins to the spindle poles suggested that 14-3-3ε could regulate spindle polarity; however, a GFP-tubulin reporter demonstrated that 14-3-3ε regulates spindle stability rather than initial polarity ^96^. Immunoprecipitation and localization assays provided evidence that 14-3-3ε stabilizes the spindle in part by recruiting Kinesin 14/Ncd, already discussed above ^96^. In this context, 14-3-3 proteins are thought to promote selective interaction between Ncd (Kinesin 14) and spindle microtubules while preventing Ncd association with non-spindle microtubules ^96^. That Ncd/Kinesin 14 localization to the spindle is impaired in 14-3-3ε depleted oocytes and rescue of spindle deficits by wild-type Ncd/Kinesin 14 but not mutant versions of Ncd/Kinesin 14 that cannot be phosphorylated (S96A) implicated 14-3-3ε in regulating Ncd/Kinesin 14 localization and activity to promote stability of the bipolar spindle ^96^.

The 14-3-3ε interaction with phosphorylated Ncd/Kinesin 14 (S96) and similar spindle phenotypes of *14-3-3ε* depletion and *Ncd/Kinesin 14* mutants hinted that 14-3-3ε binding would promote microtubule crosslinking mediated by Ncd/Kinesin 14 *.* That diminished 14-3-3ε led to dispersed Ncd/Kinesin 14 along non-spindle microtubules and to reduced Ncd/Kinesin 14 on spindle microtubules implicated an unknown factor in locally regulating Ncd/Kinesin 14 phosphorylation to limit Ncd/Kinesin 14 interactions to spindle microtubules that are proximal to chromosomes ^96^. Consistent with this model, an adjacent serine (S94) emerged as a prime site for phosphorylation, and the CPC component Aurora A/B emerged as the candidate kinase likely to fulfil this role ^97– 99^. Biochemical data and oocyte assays confirmed that phosphorylation of S94 prevents 14-3-3ε binding and allows for microtubule binding ^96^. Moreover, an S94A mutant Ncd/Kinesin 14 could rescue *ncd/kinesin 14* loss of function in transgenic rescue assays ^96^. This new mechanism appears to share features with the well-characterized Ran-Importin system used in *Xenopus* ^40^ ( Figure 2B, C). Specifically, 14-3-3 blocks Kinesin 14 interaction with non-spindle microtubules in a manner that is analogous to Importin ( Figure 2B, C). In both pathways, release of inhibition occurs proximal to the chromosomes; Ran displaces Importin, and 14-3-3 is displaced by the CPC component Aurora B ( Figure 2B, C). As mentioned above, in the mouse, a different 14-3-3 protein has been implicated in spindle morphogenesis ^94^. It is not clear whether this 14-3-3 also acts through interaction with, and regulation of, Ncd/Kinesin 14 or acts through other factors that remain to be discovered. Nonetheless, local regulation of the activity of molecular motors with microtubule bundling functions seems to play a conserved role in generating opposing forces to provide for robust spindle assembly in the absence of centrosomes and possibly to provide insurance for equal chromosome segregation between the large oocyte and tiny polar bodies produced from meiotic division.

## Conclusions

Production of a developmentally competent oocyte is essential for normal development of an individual and survival of species. The oocyte is a highly specialized and enormous cell that must retain the capacity to give rise to all of the cells that make up an embryo. Thus, compared with somatic cells, the oocyte has unique challenges, including accomplishing an asymmetric division that directs the bulk of the maternal cytoplasm to the oocyte but equally distributes the chromosomes in the absence of centrosome-based MTOCs. The recent evidence discussed in this review indicates that assembly of the meiotic spindle is orchestrated via mechanisms that involve spatially restricted cues, including factors emanating from the chromosomes, that allow kinesin motors with microtubule-organizing activity to act only on subsets of microtubule to establish balanced action of motors with opposing activities, thus substituting for functions supplied by the centrosome in mitotic cells. Coordination between cytoskeletal elements, in part through the activity of crosslinking proteins, impacts cellular and meiotic spindle morphology to ensure that the meiotic divisions and oogenesis are successful. As discussed, several mechanisms to support microtubule nucleation and spindle morphogenesis have been discovered. Much of what we understand has come from basic genetics, including targeted and forward genetic screens, and pharmacological approaches. As highlighted herein, improved genome editing and reverse genetics tools coupled with elegant *in vivo* labeling and imaging approaches have already shed significant light on this process and will continue to do so. These technological advances and those to follow will make it feasible to systematically test candidate factors to decipher their contribution to assembly of the meiotic spindle and chromosome segregation. This is a significant biological problem with clear potential to impact reproduction and fertility, as most first-trimester miscarriages in humans are associated with defects in chromosome segregation and aneuploidy.

## Abbreviations

Asp, microcephaly-associated protein; Buc, Bucky ball; CPC, chromosome passenger complex; D-TACC, *drosophila* transforming, acidic, coiled-coil containing; GVBD, germinal vesicle breakdown; Klp61F; kinesin-like protein 61F (Kinesin 5); Macf1, microtubule actin crosslinking factor 1; Mgn, Magellan; Msp, mini spindle; MTOC, microtubule-organizing center; Nabkin, nuclear actin-bundling kinesin; Ncd, non-claret disjunctional; Par1, partitioning defective 1; Ran, Ras-like nuclear; RNAi, RNA interference; Shot, short stop; Sub, subito; TOG, tumor overexpressed gene; XCTK2, *xenopus* COOH-terminal kinesin 2 (Kinesin 14); XMAP215, *xenopus* microtubule-associated protein.
